# Factors associated with the occurrence of a fall in subjects with primary open-angle glaucoma

**DOI:** 10.1186/s12886-017-0613-1

**Published:** 2017-11-25

**Authors:** Sayaka Adachi, Kenya Yuki, Sachiko Awano-Tanabe, Takeshi Ono, Hiroshi Murata, Ryo Asaoka, Kazuo Tsubota

**Affiliations:** 1 Department of Ophthalmology, Keio University School of Medicine, Shinanomachi 35, Shinjuku-ku, Tokyo, Japan; 2 Department of Ophthalmology, the University of Tokyo, Graduate School of Medicine, 7-3-1 Hongo, Bunkyo-ku, Tokyo, Japan

**Keywords:** Fall, Quality of life, Primary open-angle glaucoma

## Abstract

**Background:**

The aim of the study is to investigate risk factors for future falls in subject with primary open angle glaucoma (POAG).

**Methods:**

All participants answered the following question at their baseline ophthalmic examination: Have you had any falls in the last year? (Yes/No). All study participants answered the same question every 12 months for 3 years. The means of total deviation values in the whole, superior peripheral, superior central, inferior central, and inferior peripheral visual fields (VF) were calculated. The relationship between these mean VF measurements, and various clinical factors against patients’ future falls was analyzed using multiple linear regression.

**Results:**

Two-hundred ninety four POAG patients answered the baseline and follow-up fall questionnaires over a period of three years. Among 294 subjects, 69 patients experienced a fall during the three-year follow-up. History of falls at baseline (coefficient = 1.22), history of fear of falling at baseline (0.53), best corrected visual acuity in the worse eye (7.37), prevalence of diabetes mellitus (0.60), prevalence of systemic hypertension (0.53) were selected in the optimal model.

**Conclusions:**

Visual acuity in the worse eye, history of falls, fear of falling, diabetes mellitus, and systemic hypertension are risk factors for falling in subjects with POAG.

**Electronic supplementary material:**

The online version of this article (10.1186/s12886-017-0613-1) contains supplementary material, which is available to authorized users.

## Background

Glaucoma is a disease with progressive loss of retinal ganglion cell which proceeds to peripheral visual field loss, central visual filed loss, and vision loss. Aging is a risk factor for onset and progression of glaucoma [1]. Tham et al. estimated that in 2013, the number of people (aged 40–80 years) with glaucoma worldwide was estimated to be 64.3 million, increasing to 76.0 million in 2020 and 111.8 million in 2040 [2]. Thus, in future, a lot of elderly people may spend live with glaucomatous visual field defects.

Fall is one of the leading causes of injury and death in daily living. In 2010, 21,649 people over 65 in the United States had fatalities due to falls [3]. Fall is associated with not only injury, or death, but also hospitalization, reduced quality of life, fear of falling [4], restricted daily living, subsequent admission to nursing home, and depression [5]. Preventing falls is an emerging important issue in the world.

Visual impairment is an important risk factor for a fall. In the Singapore Malay Eye study, subjects with severe visual impairment (LogMar > =1.0) in the worse eye had a significantly higher risk of falling (odds ratio: OR =1.6; 95% 95% confidence interval: CI 1.1 to 2.3) after adjustment for co-variates [6]. However, the association between glaucoma and a risk of falling is controversial. In the Singapore Malay Eye study, having glaucoma (*n* = 21) increased the risk of falling by more than 4 fold (OR = 4.2; 95% CI 1.2–12.3) after adjustment for visual acuity [6]. Baig S. et al. reported that a history of fast glaucomatous visual field loss was significantly associated with falls (rate ratio, 2.28 per 0.5 dB/y faster; 95%CI, 1.15–4.52 db/y; *P* = 0.02), even after adjusting for confounding factors [7]. Black et al. reported that patients with glaucomatous inferior visual field loss had 1.5 times higher risk of falling [8]. We have previously reported that inferior peripheral visual field loss is associated with injurious fall in subjects with primary open angle glaucoma (POAG) [9]. However, in the blue mountain eye study, and in the Melbourne visual impairment project, glaucoma was not found to be a risk factor for falling [10, 11]. Further, Glynn et al. reported that visual field impairment in subjects with glaucoma was not associated with falls [12]. We have also shown that glaucomatous visual field loss is not associated with falls without injury [9]. Most of these studies investigated falls in elderly glaucoma patients, such as over 65 years old. However glaucoma is not only observed in the elderly, but also in middle-aged population. In Japan, the prevalence of glaucoma in people aged in their 40’s is about 2%, [13] and the association between glaucoma and risk of falling has not been thoroughly investigated in this population, at least in Japan.

The aim of the present study is to survey the incidence of falls in subjects with POAG and investigate risk factors for future falls, in a wide age range of Japanese patients.

## Methods

### Study design and subject enrolment

This was a observational study. Japanese patients between 40 and 85 years of age who visited Keio University Hospital (Tokyo, Japan), the Iidabashi Eye Clinic (Tokyo, Japan), or the Tanabe Eye Clinic (Yamanashi, Japan) between the period of May 1, 2011 and November 30, 2011 were screened for eligibility for this study.

### Baseline evaluation of subjects with glaucoma

Patients with glaucoma were consecutively screened for eligibility using a battery of ophthalmic examinations, including slit-lamp biomicroscopy, funduscopy, gonioscopy, intraocular pressure (IOP) measurements by Goldmann applanation tonometry, and visual field examination with a Humphrey visual field analyser (HFA) and the 24–2 Swedish Interactive Threshold Algorithm Standard Strategy (Carl Zeiss Meditec, Dublin, CA). The findings were analysed by T.S., and K.Y., both of whom subspecialize in glaucoma. The reliability of the findings was confirmed to be high, with less than a 20% fixation loss rate and less than a 15% false-positive rate [14].

#### Diagnostic criteria for POAG

POAG was diagnosed when three findings were present: (1) glaucomatous optic cupping, represented by notch formation, generalized cup enlargement, a senile sclerotic or myopic disc, or nerve-fibre layer defects; (2) glaucomatous visual field defects, defined according to Anderson and Patella’s criteria (a cluster of 3 or more points in the pattern deviation plot within a single hemifield [superior or inferior] with a *p* value <5%, one of which must have a *p* value <1%) [15]; and (3) an open angle observed on gonioscopy. We have not used IOP as a diagnostic criterion for POAG, but there were no eyes with IOP more than 25 mmHg in the current study.

### Exclusion criteria

Subjects were excluded, if they had an ophthalmological disease other than POAG that could potentially compromise visual acuity or contribute to visual field loss, such as age-related macular degeneration. Subjects were also excluded if they had a decimal best corrected visual acuity (BCVA) of less than 0.7, or had a mental disorder that prevented them from understanding the questionnaire, as registered by the doctor who performed informed consent. Of the POAG patients screened, 164 patients were excluded. The reasons for excluding subjects were as follows (the numbers in parentheses indicate the number of subjects excluded): younger than 40 years old (28 patients), older than 85 years old (25), refusal to participate (10), walked with a cane (12), dementia (3), low visual acuity (24), post retinal-detachment (21), diabetic retinopathy (36), bullous keratopathy (2), age-related macular degeneration (2), other ocular disease (1). As a result, 392 POAG patients were eligible for the study.

### Baseline questionnaire of fall

All study participants answered the following questionnaire in Japanese (translated) at baseline ophthalmic examination (Additional file 1) [16]:

1. Can you walk without assistance? (Yes/No)
2. Do you use a cane or any kind of walking aid? (Yes/No)
3. How long do you spend walking on average per day? (The number of minutes was recorded.)
4. Are you afraid of falling? (Not at all; Not much; Afraid; Very afraid)
5. Have you had any falls in the last year? (Yes/No) *

The definition of fall in our study was an event whereby a person comes to rest inadvertently on the ground.

6. Have you been injured by a fall in the last year? (yes/no)

Demographic information, recorded for all subjects, included age, sex, height, weight, alcohol intake (yes/no), smoking history (yes/no/previous), current and previous illnesses (e.g., systemic hypertension, diabetes mellitus, depression, brain infarction), and medical history, including oral medications such as sleeping aids, anti-hypertensive drugs, or tranquilizers.

### Follow up questionnaire of falls

All study participants answered the following question every 12 months ±1 month after the baseline questionnaire (translated from Japanese to English here, Additional file 2) [16]: “Have you had any falls in the last year? (Yes/No)”.

### Integrated binocular visual field

**Fig. 1:**
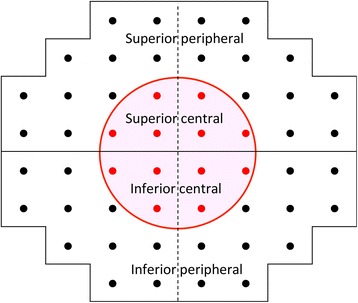
Four visual field sectors. Visual field was divided into four sectors with eccentricity, following the mapping in the 24–2 and 10–2 visual fields in the Humphrey field analyzer. The means of the TD values in each area was calculated (mTD_SP_, mTD_SC_, mTD_IC_, mTD_IP_)

A binocular integrated visual field (IVF) was calculated for each patient by merging a patient’s monocular HFA VFs, using the ‘best sensitivity’ method, where the IVF total deviation (TD) at each point was calculated using the maximum TD (least negative) value from each of the two overlapping points, as if the subject was viewing the field binocularly [17]. The IVF MD was calculated as the mean of 52 TD values across the visual field. Then, the means of the TD values in the superior peripheral, superior central, inferior central and inferior peripheral areas (mTD_SP_, mTD_SC_, mTD_IC_, mTD_IP_) were calculated, following the mapping in the 24–2 and 10–2 visual fields in the HFA (Fig. 1).

### Statistical analysis

Descriptive statistics were calculated for the demographic, medical, and visual-function variables both in patients with a history of falls and patients without history of fall. Differences were tested using the Wilcoxon test or chi square test.

The relationship between the incidence of falls and the following confounding factors were analyzed using the multivariable logistic regression model: age, sex, worse BCVA, better BCVA, mTD_SP_, mTD_SC_, mTD_IC_, mTD_IP_, body mass index, sedative or sleeping aid use, average walk minutes per day at baseline, history of diabetes mellitus, history of systemic hypertension, history of fall at baseline, and history of fear of falling. The optimal linear model was then selected among all possible combinations of 15 predictors (2^15^ patterns) using the second order bias corrected Akaike Information Criterion (AICc) index. This is because the degrees of freedom in a multivariate regression model decreases when the number of variables is large and it is therefore recommended to use model selection methods to obtain the optimal model fit by removing redundant variables [18, 19].

A *p* value less than 0.05 was considered statistically significant. Decimal visual acuity was converted to LogMAR visual acuity for analysis. All data were analysed with IBM SPSS statistics software version 21.0 (IBM Japan, Tokyo, Japan) or statistical programming language R (R version 3.1.3; The Foundation for Statistical Computing, Vienna, Austria).

## Results

Among 392 POAG patients, 294 POAG patients (75.0%) answered the baseline and follow-up fall questionnaires over a period of three years. The characteristics of these patients are summarized in Table 1. Among 294 subjects, 69 patients experienced a fall during the three-year follow-up. The incidence of falls per year was 7.8%. Subjects with incident falls were defined as the “Faller” group and subjects without incident falls were defined as the “Non-faller” group.

**Table 1:** Demographics and characteristics of POAG subjects in this study

	Number or average	Standard deviation
Number	294	
Age (years)	64.6	10.5
Gender (male/female)	169/125	57.5%/42.5%
BMI (kg/m^2^)	22.5	3.1
Prevalence of DM	44	15.0%
Prevalence of HT	89	30.3%
Better visual acuity (LogMar)	0.003	0.015
Worse visual acuity (LogMar)	0.016	0.037
mTD (dB)	−1.8	3.8
mTD_SP_ (dB)	−2.4	5.0
mTD_SC_ (dB)	−2.2	5.3
mTD_IC_ (dB)	−0.72	3.2
mTD_IP_ (dB)	−1.3	3.5
Sedative/sleeping aid use	5/10	1.7%/3.4%
Walking minutes per day	88.3	99.9
The previous fall rate	36/294 = 12.2%	–

Abbreviations: *BMI* body mass index, *DM* diabetes mellitus, *HT* hypertension, *mTD* mean of total deviation, *mTD*_*SP*_ means of the TD values in the superior peripheral area, *mTD*_*SC*_ means of the TD values in the superior central area, *mTD*_*IC*_ means of the TD values in the inferior central area, *mTD*_*IP*_ means of the TD values in the inferior peripheral area

The comparison of systemic and ocular demographic characteristics, including BCVA in the better eye and in the worse eye, MD in the better eye and in the worse eye, and systemic factors were shown in Tables 2 and 3. Patients in faller group were significantly older than patients in non-faller group. BCVA in the worse eye was significantly worse in faller group compared with that in the non-faller group. Prevalence of diabetes mellitus and systemic hypertension was significantly higher in faller group than that in non-faller group. Past history of fall, number of past falls, and history of falling was significantly associated with faller.

**Table 2:** Comparison of various systemic factors between the patients with incident fall and without incident fall

	No-faller	Faller	*P* value
Number	225	69	
Age (years)	63.9 ± 10.6	66.7 ± 10.0	0.03
Gender (male/female)	131/94 (58.2%/41.8%)	38/31 (55.1%/44.9%)	0.75
BMI (kg^2^/m)	22.3 ± 3.0	23.0 ± 3.1	0.13
Prevalence of diabetes mellitus	28 (12.4%)	16 (23.2%)	0.046
Prevalence of hypertension	61 (27.1%)	28 (40.8%)	0.048
Sedative or sleeping aid use	11 (4.9%)	3 (4.3%)	0.99
Past history of fall at baseline	18 (8.0%)	18 (26.1%)	0.0001
Number of past history of fall at baseline	0.10 ± 0.43	0.41 ± 0.83	0.0001
Past history of injurious fall at baseline	6 (2.7%)	5 (7.2%)	<0.0001
History of fear of falling at baseline	92 (40.9%)	40 (58.0%)	<0.0001
Average minutes spent walking in a day at baseline	89 ± 102	83 ± 90	0.76

Abbreviations: *BMI* body mass index

**Table 3:** Comparison of various visual factors between the patients with incident fall and without incident fall

	No-faller	Faller	*P* value
Better BCVA (LogMar)	0.0023 ± 0.012	0.0063 ± 0.024	0.12
Worse BCVA (LogMar)	0.012 ± 0.033	0.026 ± 0.047	0.005
mTD (dB)	−1.8 ± 4.0	−1.6 ± 2.9	0.56
mTD_SP_ (dB)	−2.5 ± 5.3	−2.3 ± 4.3	0.43
mTD_SC_ (dB)	−2.2 ± 5.4	−2.1 ± 5.2	0.50
mTD_IC_ (dB)	−0.8 ± 3.6	−0.5 ± 1.8	0.41
mTD_IP_ (dB)	−1.4 ± 3.7	−1.2 ± 2.5	0.73

Abbreviations: *BCVA* best corrected visual acuity, *mTD* mean of total deviation, *mTD*_*SP*_ means of the TD values in the superior peripheral area, *mTD*_*SC*_ means of the TD values in the superior central area, *mTD*_*IC*_ means of the TD values in the inferior central area, *mTD*_*IP*_ means of the TD values in the inferior peripheral area

Among the 15 variables of mTD_SP_, mTD_SC_, mTD_IC_, mTD_IP_, age, sex, body mass index (BMI), BCVA in the worse eye, BCVA in the better eye, average walking minutes in a day at baseline, prevalence of diabetes mellitus, prevalence of systemic hypertension, use of sedative and/or sleeping aid, history of fall at baseline, history of fear of falling, only a subset were included in the optimal model. These were history of falls at baseline (coefficient = 1.22), history of fear of falling at baseline (0.53), worse BCVA (7.37), prevalence of diabetes mellitus (0.60), prevalence of systemic hypertension (0.53) (see Table 4). As sum of (mTD_SP_, mTD_SC_, mTD_IC_, mTD_IP_) is identical to the mTD value of whole VF, we carried out this analysis replacing the values of mTD_SP_, mTD_SC_, mTD_IC_, mTD_IP_ with mTD of whole VF, however completely same variables were selected.

**Table 4:** The optimal model for incident fall in subjects with POAG

Variable	Coefficient	Std. Error	*P* value
History of falls at baseline	1.22	0.39	0.0016
History of fear of falling at baseline	0.53	0.30	0.076
BCVA in the worse eye. LogMar.	7.37	3.44	0.032
Prevalence of diabetes mellitus	0.60	0.37	0.11
Prevalence of systemic hypertension	0.53	0.31	0.084

Abbreviations: *POAG* primary open-angle glaucoma, *BCVA* best corrected visual acuity

## Discussion

In the current study, the incidence of falls in subjects with POAG was surveyed for three years and the risk factor for future falls was investigated. As a result, we have shown that History of falls at baseline, history of fear of falling at baseline, BCVA in the worse eye, prevalence of diabetes mellitus, prevalence of systemic hypertension were the risk factors for the occurrence of a future fall in subjects with POAG. Among the variables related to visual function, only the worse BCVA was selected as a risk factor of fall, but none of better BCVA, mTD_SP_, mTD_SC_, mTD_IC_, mTD_IP_ were selected.

In a Singapore Malay Eye study, severe visual impairment (LogMar >1.0) in the worse eye significantly increased the risk of falling (60%; OR = 1.6; 95% CI 1.1 to 2.3), but that in the better eye did not [6]. Coleman et al. also reported that women with binocular visual field loss are at greater risk of future frequent falls, but visual acuity in the better eye was not associated with falling [20]. In the Beaver Dam Eye study, Klein et al. reported that a 2.6-fold higher risk of multiple falls over 12 months, for habitual binocular visual acuity levels 0.09 logMAR or worse [21]. The Blue mountain eye study reported that visual acuity worse than 20/30 were associated with 1.9 times higher risk for 2 or more falls in a cross sectional study [10].The Blue mountain eye study also reported that incidence of bilateral visual impairment (BCVA worse than 20/40 in bilateral eye) within 5 years were more likely to report ≥2 falls in 5 years, OR 1.46, 95%CI 1.04 to 2.04 compared to participants with normal vision [22]. In the SEE study, Freeman et al. reported that binocular visual acuity was not associated with increased risk of falling [23]. Coleman et al. found that older women having a binocular visual acuity loss of 10 letters or more had an increased likelihood of falling in a prospective study [24]. Most previous studies did not investigate the effect of visual acuity in the worse eye. Wu et al. compared the posture stability by measuring the total track length and surface area of center of pressure of body sway between with one eye close or both eye open, and reported that one eye close significantly increase the posture instability [25]. These results suggest that worse BCVA in the worse eye increase posture sway, may result in increased risk of falling.

Coleman et al. reported that subjects with history of frequent falling is five times more likely to experience multiple fall in a large sample prospective study [24]. In the Salisbury eye evaluation, subjects with history of falls is two times more likely to fall in a population based prospective study [25]. In agreement with these studies, our current study suggested that previous history of falls was a risk factor for future fall in subjects with POAG.

In this study, we can’t find the association between glaucomatous visual field defect and a fall. Whether glaucoma is associated with falling is controversial [6–12, 20–27]. One possible reason for the fact that we did not observe an association between glaucoma and a fall may be attributed to the difference in the ethnicity of subjects. It has been reported that the rate of falls in Asia is lower compared to other racial/ethnicity subgroups, such as in Europe and Australia [28]. Also the hip fracture rates in Japanese people who reside in Japan and in Hawaii are half of those observed in Caucasian populations in Hawaii or in mainland USA, despite the lower bone mineral density of Japanese, which is a risk factor for fracture [29]. Recent data published from the 2011–2012 California Health Interview Survey also indicate that fall rates appear to be lower in Asians who reside in the USA compared to Caucasians and/or non-Asians [30]. Geng et al. also reported that Asian (OR 0.64, CI 0.50–0.81) and black (OR 0.73, CI 0.55–0.95) women were much less likely to have ≥1 fall in the past year, after adjusting for confounding factors [31]. Geng et al. suggested that potential reasons for these ethnicity differences may include heritable, cultural, health-related or behavioral factors that could influence risk of falling [32]. Indeed, in our previous cross-sectional study in a Japanese population, we did not find an association between glaucoma and a fall [9]. These differences by ethnicity may explain why we failed to find an association between falls and glaucomatous visual field damage.

We showed that a history of fear of falling at baseline is a risk factor for future falling. To the best of our knowledge, this is the first study that shows fear of falling is a risk factor for future falling in subjects with POAG. Fear of falling is defined as anxiety about falling and limitation of the activities in daily life [33]. Fear of falling impair balance performance, and reduce posture stability, that results in increasing risk of falling [4, 34]. Fear of falling is a psychological consequence of falling, however, subjects without history of falling could develop fear of falling. We have previously reported that severe glaucomatous visual field defect is associated with fear of falling [35], and inferior visual field loss is a predictor of future development of fear of falling (under review). Glaucomatous visual field defect may increase risk of falling via fear of falling. Thus, far of falling may be a confounder between glaucoma and falling.

Prevalence of systemic hypertension, was selected as risk factor for future falling. Tinettie et al. reported that adjusted hazard ratios for serious fall injury were 1.40 (95% CI, 1.03–1.90) in the moderate- intensity antihypertensive groups compared with non anti-hypertensive medication users [35]. However, whether anti-hypertensive drug use is a risk factor for fall or not is still in debate [36, 37]. Subjects with POAG have vascular and autonomic dysregulation [38]. The additive effect of anti-hypertensive drug on autonomic or vascular dysregulation may increase risk of falling in subjects with POAG.

Prevalence of diabetes mellitus was also chosen as a risk factor for future falling. Maurer et al. reported that subjects with diabetes mellitus is 4 times more likely to fall (adjusted HR 4.03; 95% CI,1.96–8.28) in a prospective study [39]. Several potential complications from diabetes mellitus including peripheral neuropathy, diabetic retinopathy, autonomic neuropathy manifesting as orthostatic hypotension could be potential mechanisms for falls in subjects with diabetes mellitus. The United Kingdom Prospective Diabetes Study (UKPDS) reported that tight glycemic control targeting a A1C level below 7% has also been linked to an increased risk of falls [40]. These results support our result that POAG subjects with systemic hypertension, or diabetes mellitus has higher risk of falls.

Fear of falling and previous episodes of falls are inter-correlated (*p* < 0.05, chi-square test). We included the interaction between history of falling and fear of falling, but the parameters selected in the optimal model were not changed. Also, adding this interaction resulted in the increase of AICc value by 2.1. Among other variables of age, sex, BMI, BCVA in the worse eye, BCVA in the better eye, average walking minutes in a day at baseline, BCVA in the worse eye and BCVA in the better eye had the highest correlation coefficient, but it was merely 0.60. Furthermore, adding the interaction between these BCVAs resulted in the same variables in the optimal model. Other intercorrelation had much lower values of correlation coefficient, thus the results obtained is influenced by multicollinearity among the variables.

This study is subject to several limitations. First, the self-reported questionnaire “Have you experienced a fall in the past one year” may be a source of recall bias. Second, our study did not include a control group consisting of healthy subjects. Third, we were unable to follow all the participants over the three year period; subjects who were ‘lost to follow-up’ could introduce a bias in our results. The number of drop-outs from the current study was 98 patients. These patients simply did not appear again in the clinic, and the reasons for this are not entirely clear. Although it is unlikely that the majority of these are because of injurious falls, this is still a cause of a bias in the current study. Fourth, the fear of falling measure used in this study is weak. The usage of other standardized measures of fear of falling, such as short fall efficacy scale [4], may influence our results associated with the fear of falling. Fifth, the fact that no correlation was observed with the visual field MDs might simply be due to a relatively narrow range of visual field damage in the current study. Selecting patients with various degrees of damage may uncover the role of the visual field. Sixth, our questionnaire was inherited from a previous paper [16], although this questionnaire has not gone through a proper validation process.

Finally, we did not collect the number of falls experienced in the study. It would be possible to analyze the relationship between the number of falls and glaucoma, with a longer observation period, in a future study.

## Conclusions

Worse visual acuity in the worse eye and a history of falls are risk factors for future falls in subjects with POAG. Maintaining visual acuity in subjects with POAG may reduce fall in POAG patients.

## Additional files

Additional file 1: Baseline questionnaire. Baseline Fall related questionnaire in this study (translated from Japanese to English). (DOCX 16 kb)

Additional file 2: Follow-up questionnaire. Follow-up fall related questionnaire in this study which was performed once a year (translated from Japanese to English). (DOCX 15 kb)

## Abbreviations

AICc: Corrected Akaike information criterion
BCVA: Best corrected visual acuity
BMI: Body mass index
CI: Confidence interval
HFA: A Humphrey visual field analyser
IOP: Intraocular pressure
IVF: Integrated visual field
mTDic: Means of the total deviation in the inferior central
mTDip: Means of the total deviation in the inferior peripheral
mTDsc: Means of the total deviation in the superior central
mTDsp: Means of the total deviation in the superior peripheral
OR: Odds ratio
POAG: Primary open angel glaucoma
TD: Total deviation
VF: Visual field
