# Supplementary material for: Factors associated with the occurrence of a fall in subjects with primary open-angle glaucoma
Source: BMC Ophthalmol. 2017 Nov 25;17:213. doi: 10.1186/s12886-017-0613-1 (PMC5702084; doi:10.1186/s12886-017-0613-1)
Supplement: Supplementary file 1 — Baseline questionnaire. Baseline Fall related questionnaire in this study (translated from Japanese to English). (DOCX 16 kb) [file 12886_2017_613_MOESM1_ESM.docx]

＜Questionnaire＞

Age　(　　)　　Sex　Male・Female

Weight　(　　)kg　　Height　（　　）cm

Past History：Systemic hypertension・Diabetes Mellitus・Heart disease・Liver disease・Renal disease ・Others（　　　　　）

Oral medication use:

Sleeping aids (Yes/No); Anti-hypertensive drugs (Yes/No); Tranquilizers (Yes/No)

Smoking history：Yes/No/Previous

Alcohol intake： Yes/No

1. Can you walk without assistance? (Yes/No)

2. Do you use a cane or any kind of walking aid? (Yes/No)

3. How long do you spend walking on average per day? (The number of minutes was recorded.)

4. Are you afraid of falling? (Not at all; Not much; Afraid; Very afraid)

5. Have you had any falls in the last year? (Yes/No) *

6. Have you been injured by a fall in the last year? (Yes/No)
