# Supplementary material for: Factors associated with the occurrence of a fall in subjects with primary open-angle glaucoma
Source: BMC Ophthalmol. 2017 Nov 25;17:213. doi: 10.1186/s12886-017-0613-1 (PMC5702084; doi:10.1186/s12886-017-0613-1)
Supplement: Supplementary file 2 — Follow-up questionnaire. Follow-up fall related questionnaire in this study which was performed once a year (translated from Japanese to English). (DOCX 15 kb) [file 12886_2017_613_MOESM2_ESM.docx]

＜Questionnaire＞

1. Are you afraid of falling? (Not at all; Not much; Afraid; Very afraid)

2. Have you had any falls in the last year? (Yes/No) *

3. Have you been injured by a fall in the last year? (Yes/No)
